# Aligning the unalignable: bacteriophage whole genome alignments

**DOI:** 10.1186/s12859-015-0869-5

**Published:** 2016-01-13

**Authors:** Sèverine Bérard, Annie Chateau, Nicolas Pompidor, Paul Guertin, Anne Bergeron, Krister M. Swenson

**Affiliations:** ISEM, CNRS - Univ. Montpellier, Montpellier, France; LIRMM, CNRS - Univ. Montpellier, 161 rue Ada, Montpellier, 34392 France; IBC Institut de Biologie Computationnelle, Montpellier, France; LaCIM, Université du Québec à Montréal, Montréal, Canada; Département de mathématiques, Collège André-Grasset, Montréal, Canada

**Keywords:** Bacteriophages, Whole genome alignments, Comparative genomics, Partial orders

## Abstract

**Background:**

In recent years, many studies focused on the description and comparison of large sets of related bacteriophage genomes. Due to the peculiar mosaic structure of these genomes, few informative approaches for comparing whole genomes exist: dot plots diagrams give a mostly qualitative assessment of the similarity/dissimilarity between two or more genomes, and clustering techniques are used to classify genomes. Multiple alignments are conspicuously absent from this scene. Indeed, whole genome aligners interpret lack of similarity between sequences as an indication of rearrangements, insertions, or losses. This behavior makes them ill-prepared to align bacteriophage genomes, where even closely related strains can accomplish the same biological function with highly dissimilar sequences.

**Results:**

In this paper, we propose a multiple alignment strategy that exploits functional collinearity shared by related strains of bacteriophages, and uses partial orders to capture mosaicism of sets of genomes. As classical alignments do, the computed alignments can be used to predict that genes have the same biological function, even in the absence of detectable similarity. The Alpha aligner implements these ideas in visual interactive displays, and is used to compute several examples of alignments of *Staphylococcus aureus* and *Mycobacterium* bacteriophages, involving up to 29 genomes. Using these datasets, we prove that Alpha alignments are at least as good as those computed by standard aligners. Comparison with the progressiveMauve aligner – which implements a partial order strategy, but whose alignments are linearized – shows a greatly improved interactive graphic display, while avoiding misalignments.

**Conclusions:**

Multiple alignments of whole bacteriophage genomes work, and will become an important conceptual and visual tool in comparative genomics of sets of related strains.

A python implementation of Alpha, along with installation instructions for Ubuntu and OSX, is available on bitbucket (https://bitbucket.org/thekswenson/alpha).

**Electronic supplementary material:**

The online version of this article (doi:10.1186/s12859-015-0869-5) contains supplementary material, which is available to authorized users.

## Background

The most abundant, and probably the most diverse, biological entities are bacteriophages, the viruses that infect bacteria. Helped by recent advances in sequencing, comparative studies [1–3] use dozens – even hundreds – of genomes from bacteriophages that infect single or related bacteria species. Using dot plots and clustering techniques, these studies produce meaningful clusters that share significant similarity (> 50 %).

In order to explore the relations between bacteriophages in the same cluster, it seems natural to turn to whole genome multiple alignments. One of the main features of whole genome aligners is that they take into account genome rearrangement events that scramble the order of large segments of chromosomes (see [4] for a review). All of these approaches are based on the principle that sequence similarity is a good predictor of functional similarity. And it is, most of the time. One notable exception are bacteriophage genomes, in which similar biological function may be encoded by dissimilar sequences – sequences with no detectable similarity, either as nucleotide sequences, or as amino acid sequences – encoding different protein folds, rendering traditional multiple sequence aligners mostly useless [5–7].

In this paper, our goal is to construct biologically meaningful multiple alignments of whole bacteriophage genomes from the *Siphoviridae*, the largest family of *tailed* bacteriophages [8]. In order to achieve this, we exploit unique structural properties of these genomes. The main one is that even loosely related tailed bacteriophages are often *functionally collinear*, meaning that different functionalities mostly follow the same order on the genomic sequence, which is, up to a circular permutation: lysogeny, DNA assembly, head morphology and DNA packaging, tail assembly, and lysis [9]. The second one is that genome size is constrained by the fact that it must fit into a capsid whose shape, thus volume, is geometrically determined by a handful of genes. This size constraint implies that segment duplication is a rare event within a genome, and when it occurs, duplicated sequences are short. Last, but not least, bacteriophage genomes are characterized by an *“unusually high degree of horizontal genetic exchange in their evolution”* [10], resulting in large sequences – up to thousands of base pairs – that are exact or almost exact matches between different strains.

**Fig. 1:**
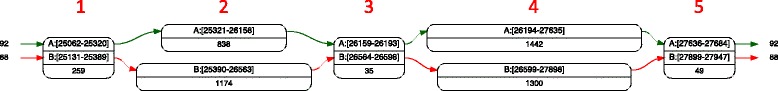
Local alignment of *S. aureus* bacteriophages 88 and 92. The first, third and fifth columns are exact matches of length 259, 35 and 49. The sequences in the second and fourth columns do not have detectable similarity, but, given their similar position along the genome, putatively encode similar biological functions. Indeed, the fourth column encodes two variants of the *integrase* gene

As an example of this last feature, Fig. 1 compares two segments of *S. aureus* bacteriophages 88 and 92 (see Table 1 for all accession numbers of bacteriophages discussed in the paper). The figure is composed of 5 columns, three of which corresponding to exact matches of length 259, 35 and 49, and the other two columns containing large segments occupying the same position but without any ‘detectable’ similarity, meaning neither the nucleotide nor the translated sequences produce any significant BLAST hit.

**Table 1:** Accession numbers and datasets

Name	Accession	Start	End	Dataset
U2	AY500152	75	45955	Myco6
Alvin	KP027205	75	45163	Myco6
DD5	EU744252	75	46027	Myco6
BillKnuckles	JN699000	75	45715	Myco6
Perseus	JN572689	75	47564	Myco6
Dreamboat	JN660814	75	44313	Myco6
U2	AY500152	1	33000	Myco29
Doom	JN153085	1	33000	Myco29
Alvin	KP027205	1	33000	Myco29
BXB1	AF271693	1	33000	Myco29
Solon	EU826470	1	33000	Myco29
Bethlehem	AY500153	1	33000	Myco29
DD5	EU744252	1	33000	Myco29
Pinto	KJ690250	1	33000	Myco29
BillKnuckles	JN699000	1	33000	Myco29
KBG	EU744248	1	33000	Myco29
Lesedi	JF937100	1	33000	Myco29
Museum	JF937103	1	33000	Myco29
Violet	JN687951	1	33000	Myco29
Kugel	JN699016	1	33000	Myco29
MrGordo	JN020140	1	33000	Myco29
KSSJEB	JF937110	1	33000	Myco29
Switzer	JF937108	1	33000	Myco29
Perseus	JN572689	1	33000	Myco29
Dreamboat	JN660814	1	33000	Myco29
Seabiscuit	KJ194585	1	33000	Myco29
Trouble	KF024724	1	33000	Myco29
BPBiebs31	JF957057	1	33000	Myco29
Wheeler	KF416340	1	33000	Myco29
Graduation	KF560331	1	33000	Myco29
JC27	JF937099	1	33000	Myco29
Thor	KP027204	1	33000	Myco29
Aeneas	JQ809703	1	33000	Myco29
SarFire	KF024726	1	33000	Myco29
SkiPole	GU247132	1	33000	Myco29
phiETA3	NC_008799	1	43282	Staph4
phiNM1	NC_008583	1	43128	Staph4
phiNM2	DQ530360	1	43145	Staph4
B236	KP893290	1	43228	Staph4
85	AY954953	1	44283	Staph6
88	AY954966	1	43231	Staph6
92	AY954967	1	42431	Staph6
29	AY954964	1	42802	Staph6
187	AY954950	1	39620	Staph6
53	AY954952	1	43883	Staph6

The mosaic patterns exhibited by the comparison of two bacteriophages, as illustrated in Fig. 1, have given rise to the *modular* theory of phage genome organization [5], which postulates that biological functions are grouped into modules whose order is mostly conserved along the genomic sequence. Each module has *variants* that perform the same function, possibly encoded by dissimilar sequences. The fact that two variants are *aligned* in the pairwise comparison may allow the transfer of functional annotation between two collinear phages.

While it is easy to do a pairwise comparison of genomes, upgrading the comparison to multiple genomes is not simple. The standard approaches used by multiple sequence aligners often start by identifying *anchors*, that are similar segments of significant length shared by all genomes, and then align the sequences between the anchors. Anchors exist in sets of related bacteriophages, but may be very short or very far apart, and the sequences between the anchors may fail to align properly.

The theory behind our framework is based on *partial order alignment graphs* [4, 11–13] which were initially developed for standard multiple sequence alignments. Most applications of these graphs require, in the last phase of the alignment, a linearization of the graph. However, an interesting suggestion appearing in [13] is to skip this last step and work directly with the partial order: this is exactly what is needed for bacteriophage genomes, but apparently the approach has yet to be applied in this context.

Among the multiple sequence aligners, progressiveMauve [14] is one of the few that recognizes the need to identify ‘local’ anchors, that are shared by a subset of the target genomes. Unfortunately, Mauve alignments are linearized, blurring the combinatorial properties of the partial order.

Our evolutionary model includes typical mutations characterizing sequence evolution such as substitutions and indels, gains and losses of functions, and recombinations. For sets of genomes that have evolved under these conditions, we will show that it is possible to identify functionally related sequences even when they lack similarity. We also detect large rearrangements events that contradict the functional collinearity hypothesis, such as gene transpositions, and duplications.

In this paper, we report the conception and implementation of Alpha (**Al**ignments of bacterio**pha**ge genomes), the first aligner specifically designed for whole bacteriophage genomes. With the help of partial order structures, Alpha captures the unique mosaic structure of bacteriophage genomes, and provides an interactive graphical interface with the generated multiple alignment. We also give a detailed comparison between Alpha and progressiveMauve [14] alignments.

## Methods

### Partial order alignment graphs and functional collinearity

This section draws heavily on multiple Whole Genome Alignment (WGA) tools and definitions. However, since we have the goal of identifying functional analogs, some classical notions of this field will have a somewhat different meaning: in these cases, we try to be precise and to underline the differences.

A *matchM* between two genome sequences *G* and *H* will be denoted *M*={*G*[*s*..*t*],*H*[*u*..*v*]}, where *s* and *u* are the start positions of the match in the respective genomes, and *t*−*s*=*v*−*u*; it asserts the equalities *G*[*s*]=*H*[*u*],…,*G*[*t*]=*H*[*v*].

We next formalize the notion of homologous positions, defining what constitutes a ‘column’ of a multiple alignment, along the lines of J. Kececioglu’s original paper [11].

Let $\mathcal {M}$ be a set of matches on genomes $\mathcal {G} = \{G_{1}, G_{2}, \ldots, G_{n}\}$ and let *p*_*ij*_ denote position *j* of genome *G*_*i*_. Two positions *p*_*ij*_ and *p*_*kl*_ are *equivalent* if there is a match in $\mathcal {M}$ that asserts that *G*_*i*_[*j*]=*G*_*k*_[*l*], or if there is a sequence of matches that links *G*_*i*_[*j*] to *G*_*k*_[*l*] through a sequence of such assertions. The equivalence class of *p*_*ij*_ is denoted *⟦p*_*ij*_*⟧*, and we will refer to it as a *column*. The *support* of a column *sp⟦p*_*ij*_*⟧* is the set of genomes that have a position in the column *⟦p*_*ij*_*⟧*.

Note that there is no requirement that a column span the complete set $\mathcal {G}$, and it usually does not in bacteriophage genomes. The notion of support generalizes the notion of *anchor* that is used in WGA, which corresponds to columns whose support is equal to $\mathcal {G}$.

**Fig. 2:**
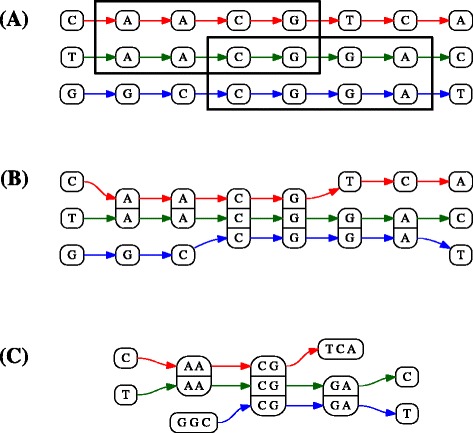
The column graph and the alignment graph. **a** A set of two exact matches on three genomes; the total length of matches is 8. **b** The matches define columns for which all positions contain the same nucleotide. The column graph is obtained by adding edges corresponding to consecutive positions in the genomes. **c** Consecutive columns with the same support are merged to form the alignment graph, whose vertices are exact alignments

The *column graph* is obtained by linking the different columns according to their order in each genome. Formally, it is the directed (multi)graph whose vertices are the columns *⟦p*_*ij*_*⟧*, and there are edges from *⟦p*_*ij*_*⟧* to *⟦p*_*kl*_*⟧* for all genomes *G*_*g*_ that have positions *p*_*gs*_ in *⟦p*_*ij*_*⟧* and *p*_*g*(*s*+1)_ in *⟦p*_*kl*_*⟧*. An example of the construction is given in Fig. 2, part (B).

#### **Definition1**.

Let $\mathcal {M}$ be a set of matches between genomes $\mathcal {G} =\{G_{1}, G_{2}, \ldots, G_{n}\}$. The set $\mathcal {G}$ is *functionally collinear with respect to*$\mathcal {M}$ if its column graph is acyclic.

In most WGA systems, collinearity is meant to represent sequences that ‘align well’. Classical collinearity is broken by pairs of dissimilar sequences, and is an indication of gene rearrangements, gains or losses, in most higher organisms. Functional collinearity, on the other hand, excludes rearrangements, but allows dissimilar sequences to be compared. Interestingly, both concepts have the same formal definition, but the use of the column graph is quite different from one application to the other.

A necessary condition for a column graph to be functionally collinear is that each column have at most one position in a given genome, since the presence of two or more positions would generate a cycle. We have therefore the following definition:

#### **Definition2**.

A set $\mathcal {M}$ of matches is said to be *valid* if, for each column *⟦p*_*ij*_*⟧*, both *⟦p*_*ij*_*⟧* and *sp⟦p*_*ij*_*⟧* have the same number of elements.

The most likely sources of invalid sets of matches are duplicated segments, and tandem repeats with a variable number of repeats, which may cause overlapping matches. Most WGA deal with these invalid matches by eliminating matches that are not unique in a genome, and by trimming overlapping matches that result from tandem repeats. Given the simple data structure that we use for the alignment graph – equivalence classes – we eliminate duplicated segments, and trim overlapping matches by post-processing the columns: any column that has more than one position for a genome is split into singletons.

Allowing the length of matches to be very small may cause misalignments, and may also create cycles in the column graph, due to random small transpositions. It is therefore wise to have a minimal length *m* for matches. In the Results and discussion section, we will discuss how the value of *m* is set, and how it can be changed.

When the column graph has no cycles, we use a condensed version, called the *alignment graph*, in which consecutive vertices with the same support are merged, as in Fig. 2, part (C).

### Construction of the alignment graph

Given a set of genomes $\mathcal {G} = \{G_{1}, G_{2}, \ldots, G_{n}\}$ we use GenomeTools [15], a suffix-array based program, to get a set of exact matches of size at least *m*. The parameter *m* is normally chosen as the minimum length such that there is no cycle in the graph. It is set by default at 15, but can be changed to any desired value. Large values of *m* are sometimes useful in initial explorations of a set of bacteriophages and small values can be used to refine alignments.

The main steps of the algorithm underlying Alpha are described in Algorithm 1. It takes as input a set of matches of minimum length *m*, constructs the column graph, and the alignment graph when possible. After initializing the graph, it uses the Union-Find algorithm [16] to construct the columns. This step runs in $\mathcal {O}(\alpha (M_{\ell })M_{\ell })$, where *M*_*ℓ*_ is the total length of matches, and *α* is the inverse of the Ackermann function [17].



The third step visits all classes, determining whether they are valid. For each class, this determination can be made in $\mathcal {O}(n)$ steps, where *n* is the number of genomes, yielding a complexity of $\mathcal {O}(nN_{\ell })$ for splitting all the non-valid classes, where *N*_*ℓ*_ is the total length of genomes. Note that at most *N*_*ℓ*_ new classes can be created by the splitting process.

In the fourth step, edges between consecutive columns are added to the graph, and it is checked for the presence of cycles that would indicate non-collinearity. This can be done in time proportional to *N*_*ℓ*_. Assuming the genomes are collinear, consecutive columns with the same support are then merged to give the alignment graph.

The total complexity is thus $\mathcal {O}(\alpha (M_{\ell })M_{\ell } + nN_{\ell })$. We will see in the Results and discussion section that the quantities *M*_*ℓ*_ and *nN*_*ℓ*_ vary greatly depending on the data.

### From the alignment graph to gapless alignments

The vertices of an alignment graph are exact alignments, since all rows are equal. Regions that do not contain exact matches of length at least *m* remain unaligned. In order to increase the compactness of whole genome alignments, we *contract* the vertices further with the following requirement:

#### **Definition3**.

The pair of vertices (*U,V*) is *contractible* if *U* precedes V in the partial order, U and V have the same support, the support of every vertex between U and V is included in *sp*(*V*), and the lengths, in base pairs, of all segments that span from U to V are the same.

A *contracted vertex* is obtained by merging into a single vertex all vertices between a pair of contractible vertices *U* and *V*, including *U* and *V*. Contracted vertices are gapless alignments, since all sequences in them have the same length. Without upper bounds to the length of contracted vertices, there is a small chance that some of these alignments have no biological foundation, since insertions and deletions could conspire in producing sequences with the same length, but low similarity. As we will see in the Results and discussion section, bacteriophage genomes are well behaved in this regard.

**Fig. 3:**
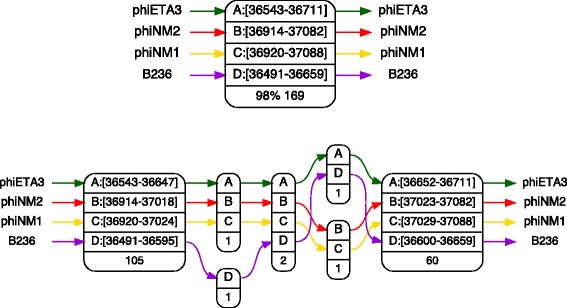
Contracted vertices. A example of a contracted vertex (top) and its expanded graph (bottom) in the alignment of four *S. aureus* bacteriophages. The length of the vertex is 169 nucleotides, and the percentage of identity is 98 %. In the expanded graph, there are two single nucleotide mutations that account for the 98 % identity

Figure 3 shows an example of a contracted vertex together with its expanded graph. All vertices display the coordinates of aligned segments, except for narrow ones, together with the common length of these segments. In addition, a contracted vertex also displays the *percentage of identity*, which is the percentage of columns that have the same conserved nucleotide in each genome of its support. Since the contracted version of the alignment graph turned out to be the most useful in practice (see the Results and discussion section), we will refer to it as the alignment graph, and use the term ‘expanded alignment graph’ when the full version is required.

## Results and discussion

In this section, we first present examples of multiple alignments and what can be deduced from them. We next assess the validity of the gapless alignments inferred by Alpha by submitting the aligned sequences to three standard aligners. Finally, we compare the results of Alpha and progressiveMauve [14] on whole genome alignments.

Alpha is an interactive tool that allows the manipulation and the visualization of whole genome alignment graphs with hundreds of vertices, involving dozens of species: we can only hope to give a glimpse of the possibilities. Alpha’s graph layouts are powered by the open source graph visualization software Graphviz [18].

### The alignment graph

The Alpha aligner, in its most basic mode, takes as input a file containing genomes in Fasta format, and produces an *alignment graph* when the genomes have no large rearranged segments. The vertices of the alignment graph are gapless multiple alignments inferred by Alpha. The vertices contain the positions of the aligned segments in each genome. They also display the length of the alignment and the percentage of columns that have the same nucleotide in each genome – for readability, vertices smaller than 20 bp may be masked. Alignments are connected by color-coded arrows, one color for each genome.

**Fig. 4:**
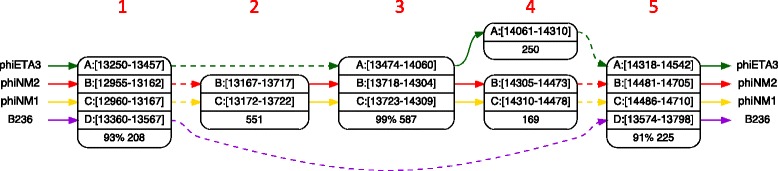
A local alignment of four *S. aureus* bacteriophages. In column 3, three phages are in a 587 bp gapless alignment, with 99 % identical columns. A major deletion in phage B236 spans columns 2, 3, and 4, and the corresponding arrow is dotted to reflect the fact that some basepairs are not shown, 6 bp in this case

Figure 4 shows an example of a local alignment of four *S. aureus* bacteriophages, in which two deletions are easily identified. Dotted arrows replace vertices that span less than 20 bp, implying that phage B236 lacks a group of functionalities that spans over a thousand bp, and phage phiETA3, one that spans over 500 bp.

Bacteriophage genomes have been sequenced for decades, and since they can adopt different linear and circular configurations in their complex life cycle, there is no universal consensus as to *where the sequence begins*. Functional collinearity is biologically defined up to a circular permutation of the sequences, but bacteriophage sequences in databases are linear. If a set of bacteriophage genomes was obtained from different projects, or different laboratories, it may be necessary to synchronize them using a simple procedure that looks for the largest similar sequence shared by all genomes, and sets the beginning there. We call this process *normalization*, and Alpha checks its input to decide whether normalization is needed.

**Fig. 5:**
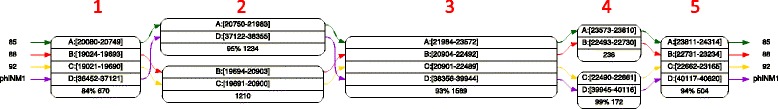
Modules and variants. Local alignment of four *S. aureus* bacteriophages clearly showing modules and variants. Notice that sequences with the same variant in the second column are switched in the fourth

Figure 5 shows a group of *S. aureus* bacteriophages whose sequences have been normalized: positions of bacteriophage phiNM1 are offset by more than 16 500 bp with respect to the other genomes, while maintaining collinearity with them. In this figure the second and fourth columns identify two pairs of large sequences. These pairs share similar *loci* within the alignment but they lack detectable similarity, and they are predicted to be variants of the same module. However, the second column splits sequences {A, D} from {B, C}, while the fourth splits sequences {A, B} from {C, D}, illustrating the very peculiar behavior of horizontal transfer in bacteriophages.

### The anchor view

Anchors are alignments that span the whole set of *n* genomes under study. They are maximal exact matches, in the sense of Hohl et al. [19], but they can be as short as 1 base pair. Each anchor is constructed as the intersection of at least *n*−1 exact pairwise alignments of length at least *m*, thus the reliability of anchors increases with both the number of genomes, and the parameter *m*. The ordered set of anchors forms the *backbone* of a set of bacteriophage genomes and captures their *common core*, as defined in Mosaic [20].

**Fig. 6:**
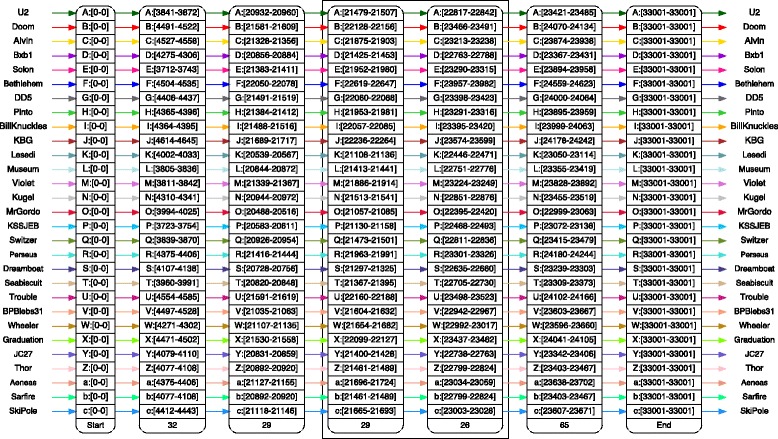
The anchor view. The alignment of 29 *Mycobacterium* bacteriophage genomes is a challenge to visual approaches. Here we use a large value of *m*=175 to display a sequence of anchors, or backbone, in a single screen. The user can then zoom quickly to a specific region, and detect, as Fig. 7 shows, interesting subsets of genomes. Boxes group pairs of anchors bounding a gapless alignment

**Fig. 7:**
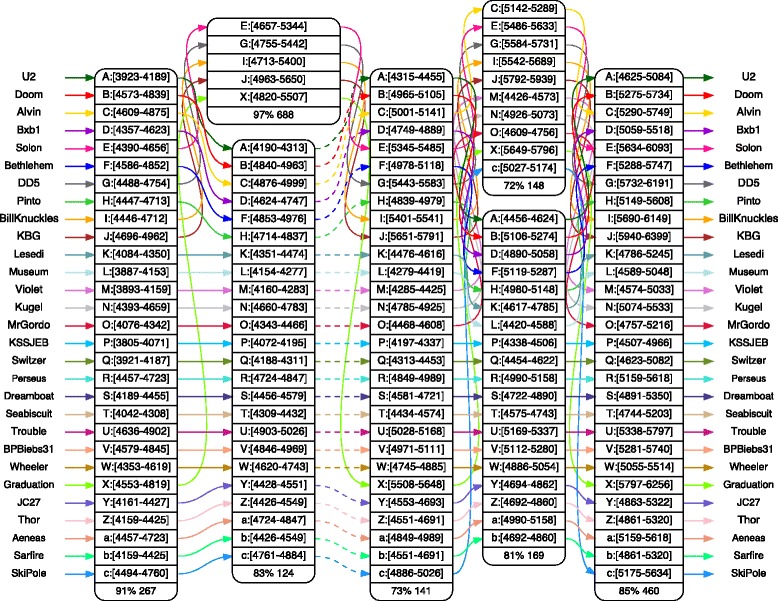
Zooming from the anchor view. Local alignment of a set of 29 *Mycobacterium* bacteriophages, obtained by zooming in from the anchor view of Fig. 6. Working with a large number of phages allows us to quickly identify subsets of phages that have distinct patterns, such as phages A, C and E (U2, Alvin and Solon) in this example

The anchor view of Alpha presents a sequence of anchors for the whole genomes, or for selected regions: it is the normal starting point to explore a set of genomes. Figure 6 shows an example of an anchor view for a set of 29 mycobacteriophages computed with *m* = 175. There are not many anchors for such a large value of *m*, but they are very well supported because each anchor is defined by at least 29 exact matches whose length is at least 175 bp. Since anchors are articulation points of the alignment graphs – removing them disconnects the graph – each group of sequences spanning from one anchor to the next can be explored separately, as we show in Fig. 7.

Once a pair of anchors is selected in the anchor view, an alignment spanning from one to the other is generated. It is possible to ask the aligner to align the sequences between them, and a new value of *m* is automatically computed, generally much smaller – or set to any desired value. In Fig. 7, for example, the value of *m* is 15.

### Assessing the validity of alpha gapless alignments

In order to assess the gapless alignments produced by Alpha, we ran it on three datasets. Table 2 presents the principal characteristics of these datasets such as *N*_*ℓ*_, the total length of genomes, the value of *m* used in the computation, and *M*_*ℓ*_, the total length of matches.

**Table 2:** Parameters of the three datasets. Column *n* is the number of genomes; *N*_*ℓ*_ is the total length of genomes; *m* is the minimal match length; *M*_*ℓ*_ is the total length of matches

Name	Hosts	*n*	*N*_*ℓ*_	*m*	*M*_*ℓ*_
Staph6	*S. aureus*	6	256 250	36	171 295
Myco6	*Mycobacterium*	6	274 292	25	407 536
Myco29	*Mycobacterium*	29	957 000	31	8 634 944

Dataset Staph6 contains 6 complete *S. aureus* bacteriophage genomes: 29, 53, 85, 88, 92 and 187. Dataset Myco29 contains 29 mycobacteriophage genomes from cluster A1 of the Actinobacteriophage Database (http://phagesdb.org). Myco6 contains bacteriophage genomes {U2, Alvin, DD5, BillKnuckles, Perseus, Dreamboat}, a subset of Myco29 chosen for diversity within cluster A1. Some of the mycobacteriophage genomes were trimmed in order to automatically run the experiments with small values of *m*; transpositions at the end of the sequences cause Alpha to increase the value of *m* (see Table 1 for details).

Alpha computed 491 gapless alignments of 2 or more sequences on these datasets. All 491 alignments were re-aligned using Clustal Omega [21], T-Coffee [22] and Muscle [23], using the default values for DNA alignments, and we report the number of alignments that include gaps. For each dataset, Table 3 gives the number of vertices in the expanded and contracted alignment graphs, the number of vertices containing at least two sequences, the number of contracted vertices, and the number of gapped alignments obtained by the three aligners.

**Table 3:** Statistics on the alignments of the three datasets

Dataset:	Staph6	Myco6	Myco29
Total number of vertices before contraction	1565	5327	10897
Total number of vertices after contraction	323	729	2324
Vertices aligning at least two sequences	202	425	964
Contracted vertices	78	154	259
Clustal Omega gapped alignments	0	1	0
T-Coffee gapped alignments	1	2	2
Muscle gapped alignments	2	5	5

All four aligners agree on 479 of the 491=78 +154+259 gapless alignments proposed by Alpha. The remaining 12 were contested by one or more of the aligners, for a total of 18 gapped alignments. They cover 8 different regions of the genomes since, in four cases, aligners proposed gapped alignments for the same region in both the Myco6 and Myco29 datasets.

Judging whether nucleotide alignments are in fact gapless is a delicate task. When proteins sequences were available, we used the gapless protein alignments to reject the corresponding gapped nucleotide alignment. This was done by using tblastx with sequences that do not share the same gap patterns – all the gapped alignments showed only two different gap patterns – and by confirming annotations using blastx. Using this method, we could rule out 12 of the 18 alternative gapped alignments, as Table 4 shows, for Regions 1, 2, 3, 4, and 8. All sequences and alignments are available in the Additional file 1.

**Table 4:** Validating gapless alignments: each column contains the number of predicted gapped alignments by each aligner. The number of mis-alignments is given in the last lines

Region’s start and end positions	Alpha	Clustal O	T-Coffee	Muscle	Gapless	Method
1. U2[1869-2931]	0	0	0	2	Yes	tblastx
2. U2[6932-7965]	0	0	0	2	Yes	tblastx
3. U2[10982-11518]	0	0	2	2	Yes	tblastx
4. U2[11521-12378]	0	0	0	2	Yes	tblastx
5. U2[17171-18327]	0	1	1	1	Yes	switch
6. U2[76-263]	0	0	1	1	?	tie
7. 85[23847-24292]	0	0	0	1	Yes	majority
8. 85[35337-35536]	0	0	1	1	Yes	tblastx
Error(s) if Alpha is right for Region 6	0	1	5	12		
Error(s) if Alpha is wrong for Region 6	1	2	4	11		

For Region 5, the three aligners proposed three different gapped alignments, when aligning the 6 genomes of dataset Myco6, but they all switched to gapless alignments when aligning the same region in the 29 genomes of dataset Myco29, which contains Myco6. Using the principle that alignments with more sequences should be more accurate, those three gapped alignments were also ruled out.

Of the remaining three gapped alignments, one was proposed by Muscle, while Alpha, Clustal Omega and T-Coffee proposed gapless alignments (Region 7). Given the overall poor performance of Muscle, we chose the rule of the majority. Finally, for Region 6, there was a tie between the four aligners, and there was no decision.

As the results of Table 4 clearly indicate, Alpha gapless alignments were confirmed in all cases except for the tie in Region 6, where Alpha and Clustal Omega – the two best gapless aligners – predict a gapless alignment against T-Coffee and Muscle, who have the most confirmed misalignments. This is a welcomed and surprising result, since the only parameter of Alpha is *m*, the minimal length of exact matches: it does not rely on thresholds, and does not maximize any score. Instead, it relies on the transitive properties of the equality relation to provide reliable anchors, and on the unique constraint imposed on bacteriophages who, before traveling, must pack their whole genome in a small suitcase.

### Comparison with Mauve alignments

In the preceding section, we showed that Alpha alignments are sound, in the sense that they predict biological meaningful similarities. The next question is to evaluate to what extent Alpha captures all meaningful similarities.

**Fig. 8:**
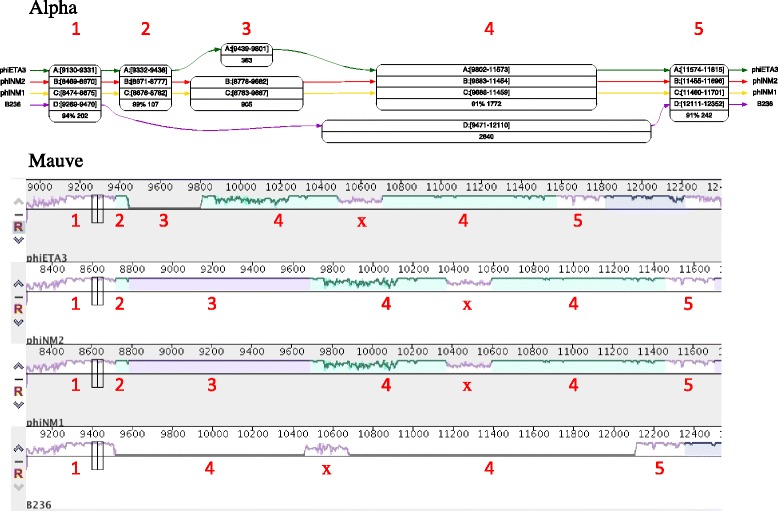
Visual comparison. Alpha and Mauve alignments of the same region of the Staph4 dataset. Numbers in red were added to show the correspondence between Alpha alignments and Mauve color blocks. All Alpha alignments are found in the Mauve alignment, but Mauve apparently detects an alignment of the four genomes that is missed by Alpha: the block labeled ‘x’. We show in the text that block x is a misalignment

In order to do this, we compared Alpha with progressiveMauve [14], since it is one of the few aligners that explicitly computes the partial order underlying collinear blocks, storing this information in the .backbone file generated during an alignment. As Fig. 8 shows, Mauve alignments are displayed in a linear way, using colors to show which subsets of segments are in an alignment. With more than a handful of genomes, such as the Myco29 dataset, this type of visualization quickly becomes impractical (see Additional file 2: Figure S1 that shows the Mauve alignment of the region spanned in Fig. 7).

Comparing partial order alignments stemming from two different aligners turned out to be a daunting task: an alignment proposed by one aligner can be broken into several alignments by the other, since alignments may involve different subsets of the input genomes. The most practical approach was to compare only alignments that included all input genomes from both aligners.

We used a dataset of four *S. aureus* phages {phiETA3, phiNM2, phiNM1, B236}, with default parameters for progressiveMauve, and interactively setting *m* between 13 and 21 for Alpha. All alignments from both aligners were identified by their phiETA3 start and end positions, and further refined by whether they were identified by Mauve only, Alpha only, or both. This produced 70 blocks, available in the Additional file 3, covering 13706 (31.7 %) of the 43282 bp of the phiETA3 genome. Of the 70 blocks, 42 were quite short, from 1 to 96 bp, while the length of the remaining 28 ranged from 123 to 3864 bp.

**Fig. 9:**
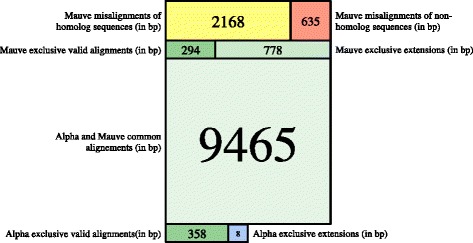
Alignments comparison. Diagram representing the repartition of 13706 bp of 70 block alignments by Alpha or Mauve. The top 5 rectangles are from alignments produced by Mauve, and the bottom 3 are from Alpha alignments. Green and blue rectangles represent valid alignments, while yellow and red are misalignments

All blocks predicted by both aligners were considered as valid alignments. Furthermore, short blocks (less than 100 bp) that were predicted by only one aligner – mostly by Mauve – were considered as valid: they averaged 24 bp. Three of the remaining large blocks – two predicted only by Mauve, and one only by Alpha – were validated using protein alignments. Figure 9 shows, in the various green-blue-shaded rectangles, the repartition of valid alignments, measured in total bp length, between common and exclusive alignments for Mauve and Alpha. These results are further partitioned by discriminating whether a block is an alignment, or an extension of an alignment.

Seven alignments predicted only by Mauve, totaling 2803 bp, were suspicious. They correspond to the yellow and red rectangles of Fig. 9. Three of them had non-significant tblastx results, despite the fact that at least one of the two sequences was annotated as coding. The remaining four alignments had a high number of gaps, contradicting the amino acid alignments of the corresponding homolog coding sequences. An example of each of these cases is detailed below, and the evidence for all others is detailed in Additional file 3.

*Example of the non-homolog case*. This is the block labeled ‘x’ in Fig. 8. Mauve aligns phage phiETA, [10484-10705], with phage B236, [10459-10678]. The nucleotide alignment has 8 gaps of lengths 1, 2, 3, 1, 3, 2, 2, and 1, over 222 bp. The best tblastx hit between the two sequences has length only 9 aa, with 4/9 identities, and with an e-value of 0.5. In this region, phage B236 is annotated for protein AKC04696:49-118, while phage phiETA3 is annotated for two proteins: YP_001004350:238-286 followed by YP_001004350:1-20. Thus there are two annotated coding regions with no detectable similarity, and trying to align them using the given nucleotide alignment would introduce numerous frameshifts.

*Example of the homolog case* (contradictory alignments). Mauve aligns phage phiETA, 8704-9129, with phage phiNM2, 8046-8468. The nucleotide alignment has 7 gaps, of lengths 2, 2, 1, 1, 3, 4, and 4. The corresponding blastp 142 aa alignment of annotated proteins YP_001004347:1-142 and ABF73110:1-141 has 58/142(41 %) identities, 92/142(64 %) positives and 1 gap. This is a rather good alignment of two distant homologs, but the Mauve alignment does not reflect the conservation of the two proteins.

When one excludes Mauve misalignments – that is, considering the green and blue rectangles of Fig. 9 – both aligners generally agree on the topology of the alignment graphs. As could be predicted, due to Mauve’s seed and extend strategy, it has wider alignments compared to Alpha, and this accounts for the 778 bp extensions that Alpha could not predict. On the other hand, the 8 bp extensions that Alpha alone predicted, in four different blocks, were in fact verified to be exact matches that Mauve somehow missed. The two aligners also failed to detect a few small alignments, with a little advantage to Alpha: Mauve detected 294 bp that Alpha did not, while Alpha detected 358 bp that Mauve did not.

The high rate of Mauve misalignments on bacteriophage genomes is problematic. More than 20 % of Mauve column alignments on this dataset are either random, or belong to nucleotide alignments whose gaps contradicts the corresponding annotated proteins alignments. It should be noted, however, that the Mauve algorithm uses Muscle to produce alignments between its own anchors, and the way Muscle introduces gaps in nucleotide alignments does not seem to be appropriate for bacteriophage genomes, as we already saw in the preceding section on validating Alpha gapless alignments.

Overall, Alpha’s alignment strategy captures the essential features of bacteriophage genomes. Regions that are similar are detected, while more dissimilar regions are not aggressively aligned. Being a conservative aligner, Alpha relies on functional collinearity to predict distant homologs that should be aligned using amino acids translations, such as the variants in columns 2 and 4 of Fig. 5.

## Conclusion

Due to their peculiar mosaic structure where similar functions do not correspond to similar sequences, bacteriophage genomes are not well-suited to traditional whole genome alignment techniques. On the other hand, they exhibit features that can be leveraged to obtain alignments, most notably functional collinearity, a low duplication rate, and the presence of long shared sequences.

In this paper, we presented a mathematical model based on partial order graphs for performing multiple alignment of bacteriophage whole genomes, along with algorithms to operate on the model. Relying exclusively on the equality relation, the model is almost parameter free, greatly reducing the need to calibrate the aligner, yet delivers biologically meaningful results. The model has been implemented in the form of an interactive aligner that can perform multiple alignments of dozens of genomes and present the result in an attractive format.

We also showed that Alpha, used on bacteriophage genomes, produces biologically meaningful alignments, while avoiding the high rate of misalignments of complex heuristics such as progressiveMauve.

Our model supposes that all genomes under consideration are functionally collinear. This is often the case, but not always. Our program can detect when this condition is not satisfied – indicating the presence of rearrangements – but does not perform the alignment in such a case. A short-term goal is to extend our mathematical model and aligner to deal with rearrangements.

Some bacteriophage genomes present in the online databases are well annotated, other are less so. Another goal is to extend the aligner in order to perform automated transfer of annotations using the generated alignments.

Finally, while we focussed this study on Siphoviridae, we plan to test Alpha on more general viruse families in which horizontal transfer is widespread, and for which the collinearity property may hold.

The code, along with installation instructions for Ubuntu and OSX, is available on bitbucket (https://bitbucket.org/thekswenson/alpha).

## Additional files

Additional file 1
**Regions in which gapless alignments predicted by Alpha are contested by Clustal Omega, T-Coffee or Muscle.** (ZIP 32 kb)

Additional file 2
**Mauve alignment corresponding to the Alpha alignment of Fig.** 7 **.** (JPG 962 kb)

Additional file 3
**Detailed comparison of Alpha and Mauve alignments for the four** ***S. aureus*** **phages phiETA3, phiNM2, phiNM1 and B236.** (XLSX 83 kb)
