# Supplementary figures and images for: Aligning the unalignable: bacteriophage whole genome alignments
Source: BMC Bioinformatics. 2016 Jan 13;17:30. doi: 10.1186/s12859-015-0869-5 (PMC4711071; doi:10.1186/s12859-015-0869-5)

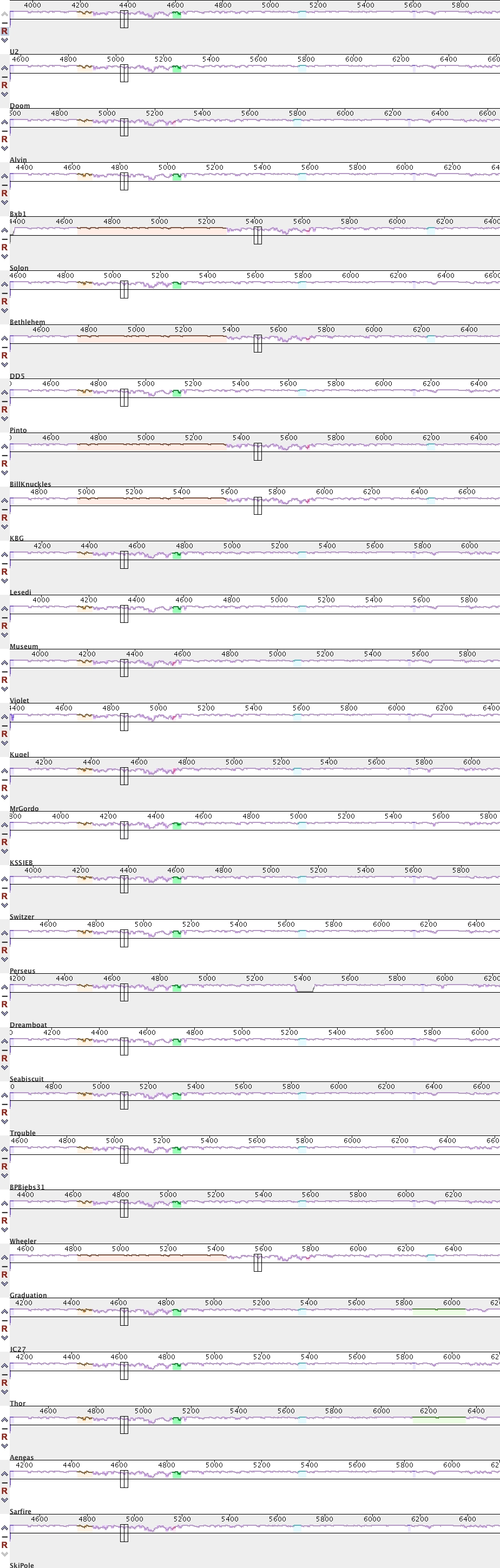

Supplement: Additional file 2 — Mauve alignment corresponding to the Alpha alignment of Fig. 7 . (JPG 962 kb) [file 12859_2015_869_MOESM2_ESM.jpg]
